# Unveiling InTe for flexible thermoelectric applications with enhanced performance via Bi/Se co-doping and MnO₂ integration

**DOI:** 10.1038/s41598-026-35782-1

**Published:** 2026-01-17

**Authors:** Manasa R Shankar, A. N. Prabhu, Ramakrishna Nayak

**Affiliations:** 1 Department of Physics, Manipal Institute of Technology, Manipal Academy of Higher Education, Manipal, 576104 India; 2 Department of Humanities and Management, Manipal Institute of Technology, Manipal Academy of Higher Education, Manipal, Karnataka 576104 India

**Keywords:** Indium telluride, Co-doping, FTEG, Screen printing, Power output, Energy science and technology, Materials science, Nanoscience and technology, Physics

## Abstract

Conventional thermoelectric materials are limited by rigidity, high synthesis costs, and poor compatibility with flexible devices. Despite progress, the development of novel, low-cost, and scalable materials for flexible thermoelectrics remains limited. The novelty of this work lies in introducing InTe as a printable thermoelectric material and demonstrating the first screen-printed flexible thermoelectric generators (FTEGs) based on InTe. Pristine and Bi/Se co-doped InTe were synthesised via solid-state reaction and fabricated through a cost-effective, scalable screen-printing method. Co-doping effectively tuned the crystallinity, carrier concentration, mobility, and band structure. Among the co-doped samples, In_0.94_Bi_0.06_Te_0.97_Se_0.03_ achieved a Seebeck coefficient of ~ 1320 µV/K and showed a maximum power output of ~ 29.45 nW at a temperature gradient of 100 K. The other novelty of this work is the incorporation of MnO₂ to form a printed p–n heterojunction, which improves the conductive pathway, leading to a peak power output of 48.41 nW, approximately 1.64 times higher than that of the In_0.94_Bi_0.06_Te_0.97_Se_0.03_ sample. The FTEGs exhibited approximately 2% resistance variation after 500 bending cycles and at various angles, confirming excellent mechanical durability. This work establishes InTe as a promising printable thermoelectric material and highlights co-doping and MnO_2_ incorporation as powerful strategies for flexible energy harvesting.

## Introduction

The escalating global energy crisis, compounded by the rapid depletion of fossil fuel reserves and mounting environmental concerns, has intensified the pursuit of sustainable and eco-friendly energy conversion technologies^1^. Recognising this, the United Nations has outlined 17 Sustainable Development Goals (SDGs), among which Goal 7 targets universal access to affordable, reliable, and modern energy. Despite significant progress in renewable energy technologies, enhancing the efficiency of energy generation and utilisation remains a key challenge. Alarmingly, it is estimated that over 60% of the primary energy produced worldwide is dissipated as waste heat during generation, conversion, and consumption processes, particularly in sectors such as manufacturing, transportation, and power generation^2^. This substantial thermal loss highlights an underutilised opportunity for energy recovery through thermoelectric generators (TEGs), which facilitate the direct conversion of temperature gradients into electrical power via the Seebeck effect^3^. Owing to their solid-state nature, absence of moving parts, long operational lifespans, and broad adaptability to diverse temperature regimes, TEGs have emerged as a compelling solution for boosting energy efficiency and supporting the transition toward a more sustainable energy future^4^.

In parallel, the rapid advancement of wearable electronics and the Internet of Things (IoT) has created a growing demand for lightweight, autonomous, and self-powered devices^5^. At the same time, widely used conventional batteries suffer from limited operational lifetimes, rigidity, safety risks, and the need for periodic replacement or recharging, especially in remote or embedded systems^6,7^. In contrast, flexible thermoelectric generators (FTEGs) offer a sustainable alternative by continuously harvesting body or environmental heat and converting it into electrical energy without moving parts^8^. Their silent operation, longevity, and maintenance-free performance make them ideal for applications in wearable health monitors, smart textiles, soft robotics, distributed sensor networks, and low-power electronics^9^.

Several high-performance inorganic thermoelectric materials have been extensively investigated for use in flexible thermoelectric generators (FTEGs). Traditional materials such as Bi₂Te₃^10^, PbTe^11^, GeTe^12^, and SnTe^13^ have demonstrated excellent thermoelectric performance owing to their favourable electrical transport properties and intrinsically low lattice thermal conductivity. However, their intrinsic brittleness, toxicity, and the requirement for high processing temperatures pose significant challenges for integration with flexible substrates and scalable fabrication techniques. Bi₂Te₃ remains the benchmark material for near-room-temperature applications, yet its high tellurium content limits large-scale deployment. PbTe offers excellent thermoelectric efficiency, but concerns about lead toxicity restrict its practical use^14^. SnTe and GeTe have emerged as promising, environmentally benign alternatives; however, their relatively high intrinsic carrier concentration and lattice thermal conductivity continue to limit overall thermoelectric performance^15^. In this context, indium telluride (InTe) has emerged as a promising yet underexplored candidate. InTe is a mixed-valence binary Zintl chalcogenide with a tetragonal *I4/mcm* space group, characterised by ultralow lattice thermal conductivity^16^. This originates from strong anharmonicity induced by weakly bound In⁺ ions with stereoactive lone pairs, resulting in phonon-glass, electron-crystal-like behaviour^17^. Although this significantly suppresses phonon transport, InTe suffers from several intrinsic challenges, including poor electrical conductivity, low carrier mobility, and suboptimal band structure, all of which collectively limit its thermoelectric figure of merit (*ZT*). These electrical limitations arise from the mixed valence states of indium, which lead to carrier localization and hinder effective charge transport. Consequently, despite its favourable thermal properties, pristine InTe exhibits low overall thermoelectric performance. To address these challenges, reported studies have explored various elemental doping strategies, using Sb^18^, Pb^19^, Ga^16^, Cd^20^, Cu^17^, Na^17^, and alloying with CuInTe₂^21^ to improve its electrical performance. In our prior work as well^22^, we demonstrated the effectiveness of co-doping approaches in enhancing the electrical properties of InTe. However, many doping combinations and their impact on the electronic structure and transport mechanisms remain underexplored. Moreover, InTe’s potential in flexible thermoelectric applications is yet to be realised, presenting a clear research gap and the need for advanced processing and scalable, low-temperature fabrication techniques. Herein, we pioneer the fabrication of flexible thermoelectric generators based on InTe using a scalable screen printing method. This approach enables low-cost, large-area, and room-temperature processing, addressing the fabrication challenges associated with brittle and complex inorganic thermoelectric materials, and unlocking new pathways for integrating high-performance thermoelectrics into flexible and wearable technologies^23,24^.

In the present study, InTe and Bi/Se co-doped InTe powders were synthesised using the solid-state reaction method to obtain phase-pure material with controlled stoichiometry. We fabricated flexible thermoelectric films via the screen-printing technique, a scalable, cost-effective, and ambient-condition process suitable for flexible substrates. To the best of our knowledge, this work represents the first report on InTe-based flexible thermoelectric generators (FTEGs). By integrating Bi/Se co-doping to engineer the band structure and enhance carrier transport, and also incorporating MnO₂ to form efficient p–n junctions, we achieved a significant improvement in thermoelectric performance. This pioneering effort not only introduces InTe as a viable candidate for flexible thermoelectric devices but also establishes a versatile fabrication strategy for future energy harvesting applications.

## Materials employed and methodological framework

### Materials

High-purity elemental powders of tellurium (Te, 99.999%), selenium (Se, 99.999%), bismuth (Bi, 99.999%), and indium (In, 99.9%) were procured from Thermo Fisher Scientific (USA) and utilised as starting materials. Diacetone alcohol (DAA) and cellulose acetate propionate (CAP), supplied by Sigma-Aldrich, functioned as the solvent and polymeric binder in formulating the screen-printing ink. Commercial manganese dioxide (MnO₂) powder (Loba) was employed directly without additional processing. The flexible thermoelectric generator (FTEG) was fabricated on a transparent polyethylene terephthalate (PET) substrate with a thickness of 100 μm, sourced from Venlon Polyester Film Ltd, India. Screen-printable silver conductive ink (Loctite ECI 1010 E&C, Henkel, India) was applied for electrode deposition to ensure stable and reliable electrical contact.

### Synthesis approach and characterisation of pure and Bi/Se co-doped InTe materials

**Fig. 1:**
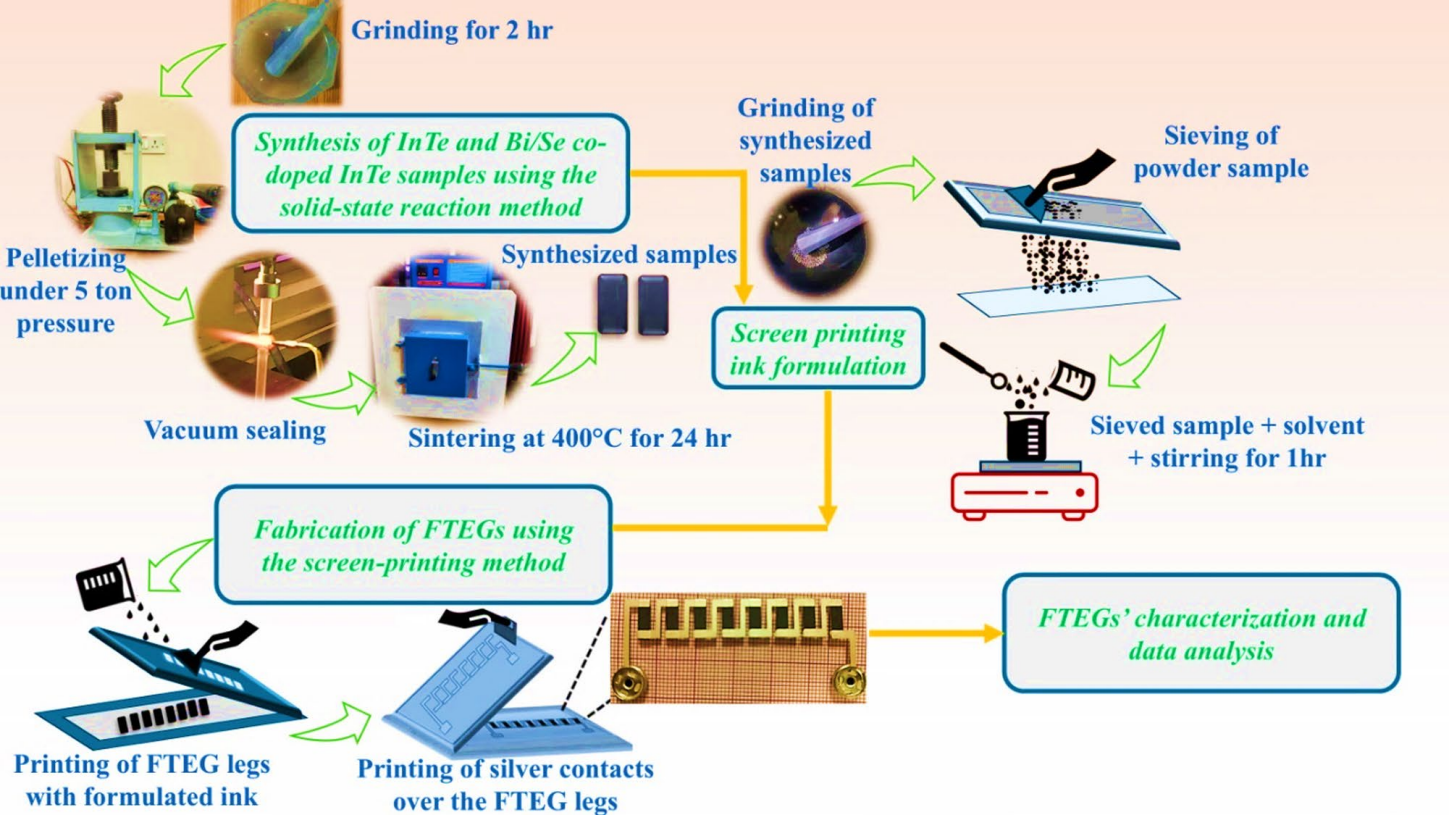
Schematic illustration of fabrication steps of InTe-based FTEGs, including synthesis, ink preparation, screen printing, and characterisation.

Pristine InTe and Bi/Se co-doped samples with compositions In_1 − x_Bi_x_Te_0.97_Se_0.03_ (x = 0, 0.02, 0.04, and 0.06) were synthesised via a solid-state reaction method, following the procedure described in our previous work^22^. High-purity elemental powders of indium (99.9%), bismuth, selenium, and tellurium (all 99.999%) were accurately weighed in stoichiometric ratios to obtain 5 g of each formulation, as summarised in Table 1. The powders were mixed thoroughly by manual grinding in an agate mortar for two hours to ensure compositional uniformity. The blended powders were compacted into pellets using a hydraulic press under a pressure of 5 tons. These pellets were sealed in quartz tubes under high vacuum (10^− 4^ Torr) to prevent oxidation and contamination. The thermal treatment was carried out at 400 °C for 24 h, followed by natural cooling inside the furnace to promote phase formation. A schematic of the synthesis process is presented in Fig. 1. The phase purity and crystal structure of the synthesised samples were previously confirmed via X-ray diffraction (XRD) and showed no secondary phases, consistent with standard tetragonal InTe patterns. As the XRD patterns obtained in this study were nearly identical to those previously reported^22^, the data are not reproduced here. The detailed thermoelectric characterisation of these bulk compositions was already reported in our earlier work^22^. Therefore, the present study adopts only the synthesis procedure from Ref. 22 for developing the printed FTEG devices.

### Preparation and characterisation of screen-printable inks for pristine and Bi/Se co-doped InTe

Following the synthesis of bulk InTe and Bi/Se co-doped InTe samples via the solid-state reaction method, the materials were ground and finely sieved to obtain uniform particle sizes suitable for ink formulation. To prepare the screen-printable inks, 15 wt% cellulose acetate propionate (CAP) was dissolved in 85 wt% diacetone alcohol (DAA), serving as the ink vehicle. Table 1 summarises the composition of the distinct Bi/Se co-doped In_1 − x_Bi_x_Te_0.97_Se_0.03_ (x = 0, 0.02, 0.04,0.06) ink formulations. The sieved powders were then mixed with the ink vehicle and homogenised using a magnetic stirrer for 1 h to ensure uniform dispersion. The viscosity of the resulting inks was carefully adjusted within the optimal range of 1750–2000 cP^25^ to facilitate smooth and consistent deposition during screen printing. The crystallographic structure of the inks was analysed using X-ray diffraction (XRD, Rigaku Ultima IV) across a scanning range of 10°–90° with a step size of 0.02°/min to assess phase purity and crystallinity. The surface morphology and particle distribution were characterised using scanning electron microscopy (FESEM, Carl Zeiss Sigma).

**Table 1:** Composition of InTe, Bi/Se Co-Doped InTe, and MnO_2_ Inks.

Ink name	In (g)	Te (g)	Bi (g)	Se (g)	MnO_2_ (g)	Ink vehicle (g)
InTe	2.368	2.631	–	–	–	1.625
In_0.98_Bi_0.02_Te_0.97_Se_0.03_	2.316	2.548	0.086	0.048	–	1.625
In_0.96_Bi_0.04_Te_0.97_Se_0.03_	2.252	2.528	0.048	0.170	–	1.625
In_0.94_Bi_0.06_Te_0.97_Se_0.03_	2.188	2.509	0.254	0.048	–	1.625
MnO_2_	–	–	–	–	5.0	1.250

### Procedure for screen printing

The fabrication of flexible thermoelectric generators (FTEGs) was achieved using a customised screen-printing technique. A formulated thermoelectric ink was carefully dispensed onto a mesh screen containing a predefined pattern. The ink was uniformly spread over the stencil using a rubber squeegee, which applied consistent mechanical pressure to transfer the ink through the open mesh regions onto a polyethylene terephthalate (PET) substrate, following the stencil geometry. The printed substrates were allowed to dry under ambient conditions to facilitate partial solvent evaporation after deposition. To enhance the structural integrity of the printed films and ensure complete removal of residual solvents, the samples were thermally cured in a hot-air oven at 60 °C for one hour. This temperature was selected to remain well below the thermal deformation threshold of the PET substrate, which typically softens above 120 °C. Although the curing temperature is low, the majority of the CAP and DAA binder components evaporated during this step. If a heat-resistant PI substrate were used, higher-temperature annealing could remove residual organics more effectively. However, such temperatures may induce thermal stress or phase changes; hence, PET was chosen to maintain flexibility and low-temperature processing compatibility. The methodology used here draws from our earlier screen-printing approaches^26,27^.

### Fabrication of printed ink films for hall measurement and flexible thermoelectric devices

Hall effect test samples were fabricated using the formulated inks to investigate the electrical properties. A high-precision screen mesh (mesh count of 120/cm, Shebro^®^, India) and stencils were designed in Inkscape^®^ software, featuring 10 mm × 10 mm square patterns. Both direct and indirect methods were used to create the stencils, ensuring accurate and consistent pattern transfer onto the substrate. The formulated inks were screen printed onto flexible PET substrates using a flat, wedge-shaped rubber squeegee (hardness: 75 Shore A), applying 12 sequential layers to achieve uniform film thickness. The printed samples were dried and later measured for thickness using a high-precision Mitutoyo 547 − 301 gauge. Hall effect measurements were conducted at room temperature using the van der Pauw method with a Keithley 6220 current source, and by applying a 0.6 T magnetic field and 50 mA current to evaluate key parameters such as carrier concentration and mobility. Each measurement was repeated three times, and the reported values are reproducible within an experimental error of ± 5%.

The primary focus of this study was the development of flexible thermoelectric generators (FTEGs) using specially formulated inks. Each FTEG consisted of eight thermoelectric legs with 3 mm × 10 mm dimensions (12 overprints), printed onto a 100 μm-thick flexible PET substrate (Fig. 1). For p–n type FTEGs, a previously optimised n-type MnO₂ ink with verified thermoelectric properties^26^ was used in combination with the p-type InTe-based inks. Commercial silver ink was used to print the interconnecting electrodes, with four layers applied to ensure high conductivity. All FTEG devices were fabricated with consistent dimensions of 15 mm × 55 mm, allowing for reliable performance comparison and evaluation.

### Characterisation of FTEG

A customised measurement system reported in our earlier work was employed to evaluate the electrical resistance, Seebeck coefficient and power output of FTEGs printed with formulated inks^26^. The setup consist of a pair of K-type digital thermocouples (Lutron TM-902 C) and a Keithley 2001 source meter for precise voltage and resistance measurements. A thermal gradient was applied along the device by heating one end of the FTEG (hot side, up to 400 K) while maintaining the other end at ambient temperature (cold side, 300 K). Measurements were performed for multiple temperature gradients with ΔT ranging from smaller values up to a maximum of 100 K. The current flows from the hot side to the cold side of the FTEG, aligned with the applied temperature gradient. The Keithley 2001 source meter recorded the thermally induced voltage across the device and its internal resistance under steady-state conditions, following established literature works^25,27–31^. The FTEGs’ mechanical flexibility was further assessed under varying bending angles using a two-probe multimeter (Fluke 179) and a custom-designed bending apparatus used in our earlier study^26^. Devices were subjected to progressive single-fold bending cycles (0, 100, 200, 300, 400, and 500) to evaluate their structural reliability and functional endurance under mechanical deformation. Figure 1 shows the detailed methodology and workflow of the present work.

## Results and discussion

### XRD analysis of prepared samples

**Fig. 2:**
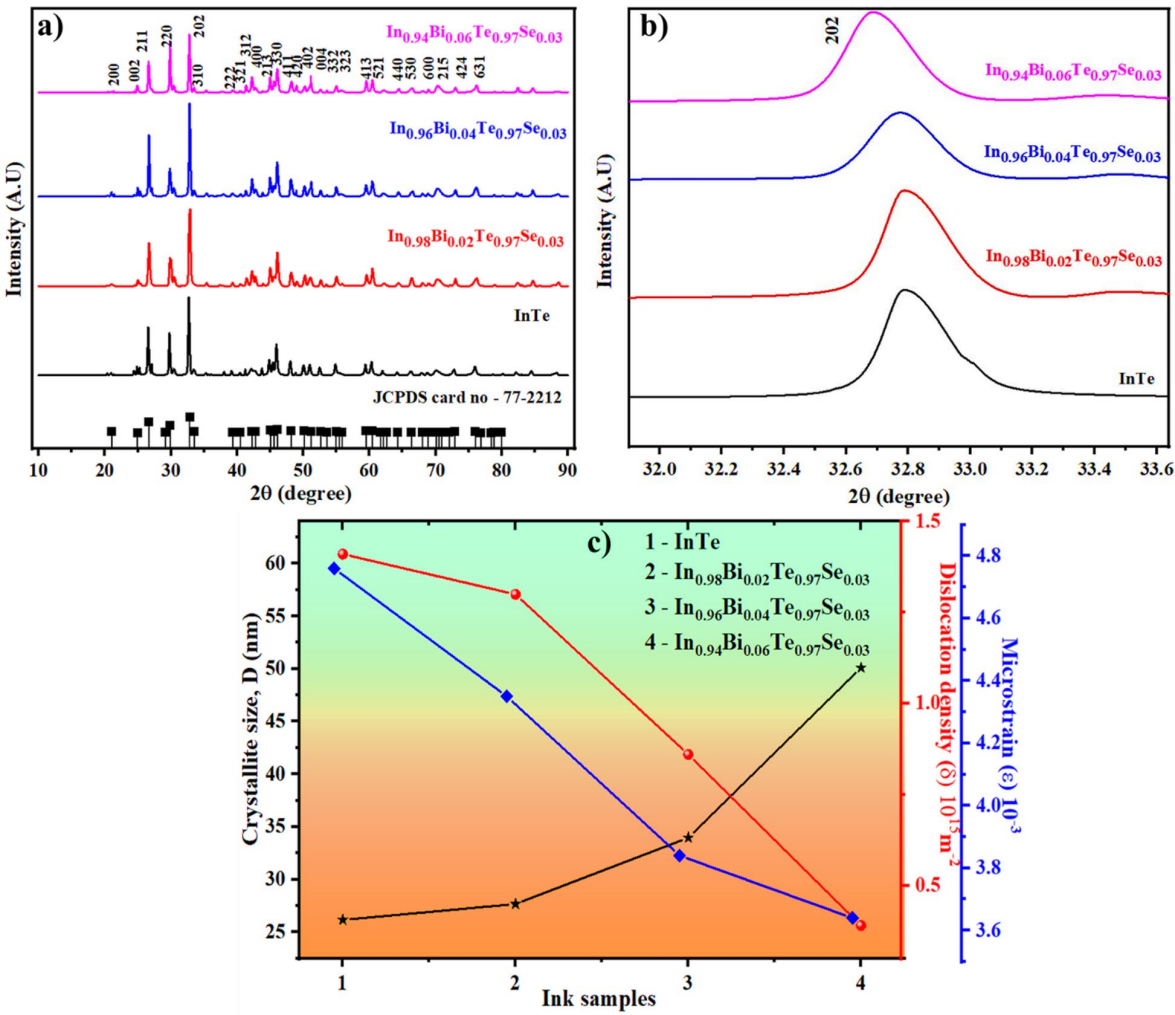
(**a**) XRD peak pattern, (**b**) shift in XRD peak pattern ((202) plane), (**c**) Plot of *D* Vs *δ* and *ε* of InTe and Bi/Se co-doped InTe ink samples.

X-ray diffraction (XRD) was used to analyse the structural properties of the InTe and In_1 − x_Bi_x_Te_0.97_Se_0.03_ (x = 0, 0.02, 0.04, 0.06) ink samples (Fig. 2a). All samples exhibit diffraction peaks that correspond to the standard tetragonal InTe phase (*I4/mcm*, JCPDS 77-2212)^20^, confirming phase purity and successful Bi/Se incorporation without secondary phases. The prominent reflections at 26.6°, 29.9°, and 32.8°, assigned to the (211), (220), and (202) planes, indicate good crystallinity and preservation of the InTe framework.

Interestingly, a non-monotonic change in peak intensities with increasing Bi concentration, particularly for the (211) and (220) planes. The (220) peak intensity initially decreases but increases again at 6% Bi concentration, whereas the (211) peak exhibits the opposite trend. These anomalies, absent in bulk InTe samples^22^, likely arise from dopant-induced changes in crystallographic texture and grain orientation. Because Bi has a larger atomic radius than In, its substitution perturbs the local lattice environment by inducing elastic strain fields and slight bond-length distortions. These strain effects can alter the surface energy of different crystallographic planes, thereby influencing their relative growth rates during crystallisation. As a result, specific planes may become energetically favourable or suppressed at different Bi concentrations, producing the observed variations in XRD peak intensities. Additionally, the organic binder system used in ink formulation may leave transient residues or induce differential shrinkage during drying and annealing, promoting preferential alignment. Such effects alter the diffracted intensity without changing phase identity^32^.

An enlarged view of the (202) peak is shown in Fig. 2b. In the Bi- and Se-doped InTe samples, this peak gradually shifts toward lower angles, indicating an increase in lattice parameters caused by the incorporation of these elements into the host matrix. The peak shifts more as the dopant amount increases, showing that the new atoms are successfully replacing the original ones in the lattice. This expansion occurs because Bi³⁺ (130 pm) has a much larger ionic radius than In⁺ (80 pm).

The observed intensity anomalies in the XRD patterns reflect underlying microstructural changes, prompting a detailed analysis of crystallite size, microstrain, and dislocation density to understand the effects of Bi doping. The crystallite size (*D*) and lattice strain (*ε*) were estimated using the Scherrer method, as described by Eq. (1)^33^,

1 $$\:D=\frac{0.9\lambda\:}{\beta\:cos\theta\:}$$

This calculation takes into account the X-ray wavelength (*λ*), the full width at half maximum (*β*), and the diffraction angle (*θ*) expressed in radians. The Scherrer method provides approximate crystallite sizes and is used here primarily to compare relative trends among pristine and doped samples measured under identical experimental conditions. Dislocations represent structural defects or irregularities within the crystal lattice. The dislocation density (*δ*) is calculated using Eq. (2)^33^,

2 $${{\delta }} = \frac{1}{{D^{2} }}$$

Likewise, the average microstrain (*ε*) within the samples was calculated using Eq. (3)^33^,

3 $$\varepsilon ~ = \frac{\beta }{{4~\tan \theta }}$$

As shown in Fig. 2c, the average crystallite size increases steadily from 26.61 nm in the pristine InTe to 50.10 nm in the 6% Bi-doped sample, indicating progressive grain growth and improved crystallinity. Concurrently, the dislocation density decreases from 1.41 × 10¹⁵ m^− 2^ to 0.39 × 10¹⁵ m^− 2^, and the microstrain reduces from 4.76 × 10^− 3^ to 3.64 × 10^− 3^. These trends indicate lattice relaxation and reduced defect density with higher Bi content, correlating directly with improved grain connectivity observed in FESEM.

### FESEM and EDAX analysis of screen-printed ink films

**Fig. 3:**
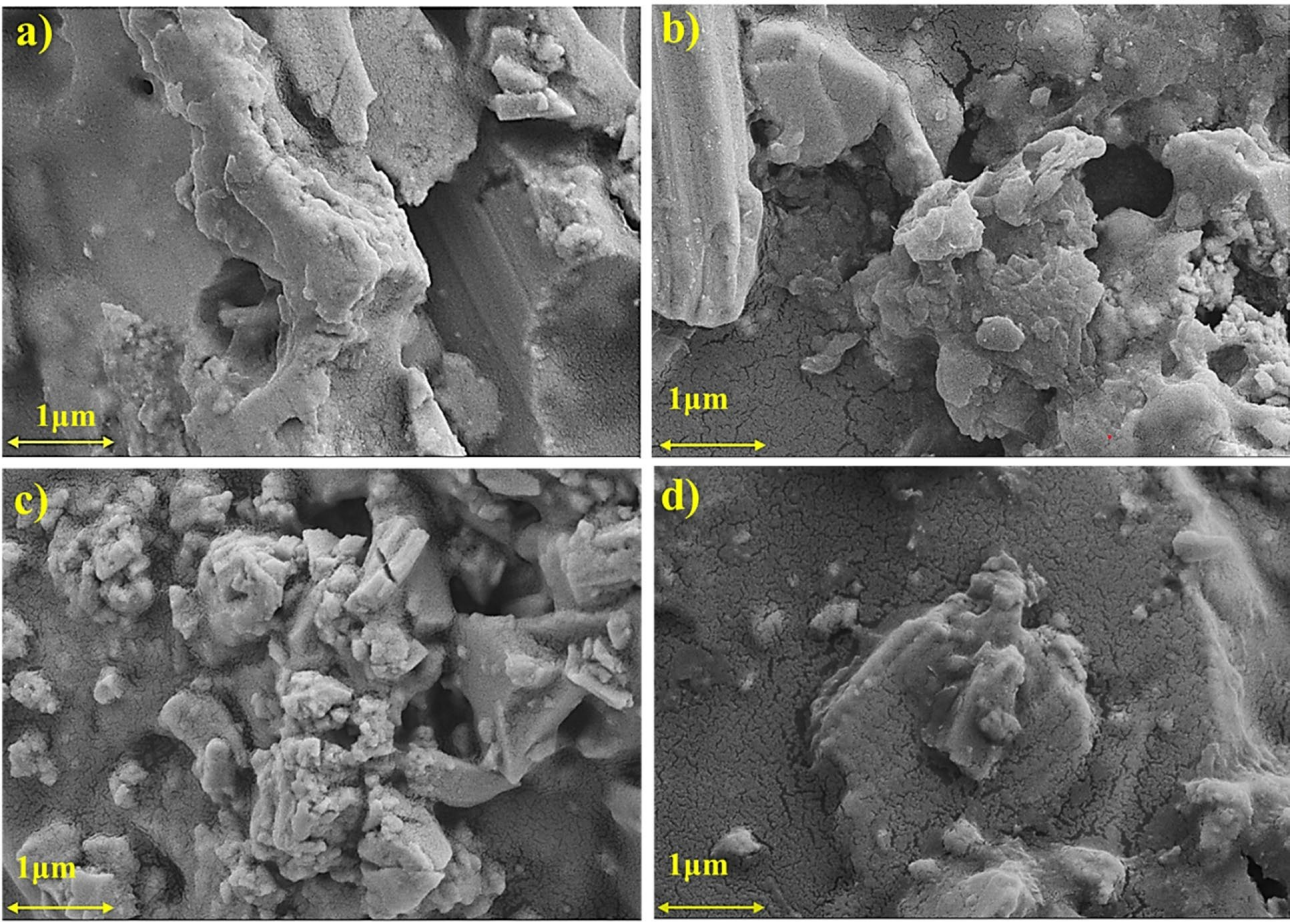
FESEM images of (**a**) InTe, (**b**) In_0.98_Bi_0.02_Te_0.97_Se_0.03_, (**c**) In_0.96_Bi_0.04_Te_0.97_Se_0.03_, (**d**) In_0.94_Bi_0.06_Te_0.97_Se_0.03_ ink film samples.

Figure 3 shows the surface morphology of InTe and Bi/Se co-doped InTe ink films observed through field emission scanning electron microscopy (FESEM). Images (a-d) correspond to pristine InTe and co-doped compositions with increasing Bi content (x = 0, 0.02, 0.04, 0.06), respectively. The pristine InTe film exhibits relatively large, layered grains with distinct boundaries and interparticle voids, reflecting limited densification. With increasing Bi content, the surface morphology becomes more fragmented and granular, particularly at a doping level of 4 at%, indicating dopant-induced grain refinement driven by lattice strain. At 6 at%, the film appears more compact with smoother textures and fewer voids, suggesting improved grain coalescence. These observations are supported by ImageJ-based porosity analysis, which shows porosity values of 11.1% for pristine InTe and 16.71%, 14.37%, and 5.29% for 2%, 4%, and 6% Bi-doped films, respectively. All samples exhibit porosity below 20%.

In addition to dopant effects, the organic binder used in ink formulation plays a crucial role in directing this microstructural evolution^34^. The binder assists in particle dispersion and rearrangement during drying and thermal processing, leading to enhanced grain connectivity, reduced porosity, and better interfacial contact. These effects improve charge transport by minimising grain boundary scattering, while the finer grain structure continues to support phonon scattering, both of which are vital for optimising thermoelectric performance^35^. These morphological observations align well with the XRD results, which reveal a progressive increase in crystallite size and a corresponding reduction in dislocation density as Bi concentration increases. The combined effect of dopant engineering and binder-mediated film formation enables favourable control over both carrier and phonon transport, thus supporting improved thermoelectric behaviour in flexible printed InTe-based devices.

**Fig. 4:**
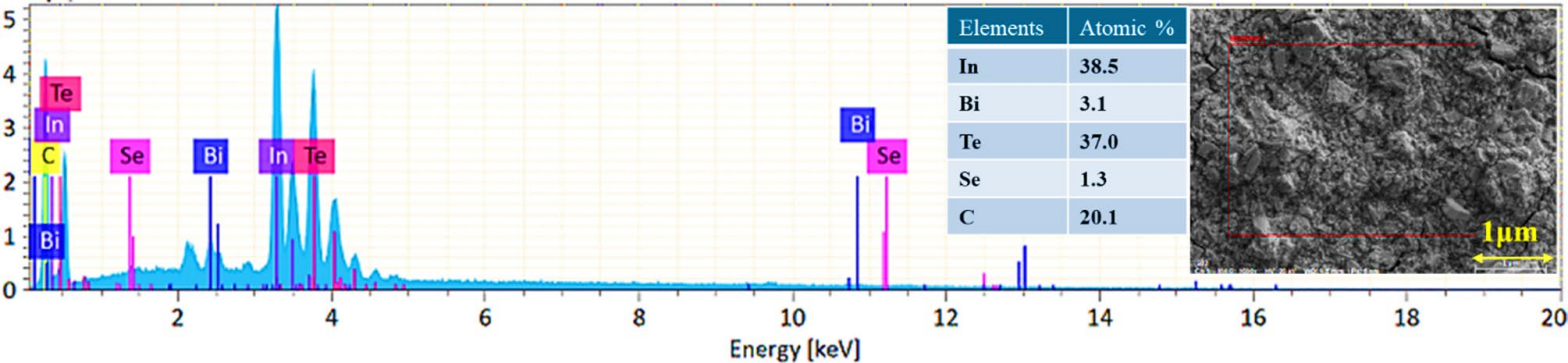
EDAX mapping of the In_0.94_Bi_0.06_Te_0.97_Se_0.03_ ink film with spectrum collection location.

Figure 4 presents the EDAX spectrum and corresponding FESEM inset image of the In_0.94_Bi_0.06_Te_0.97_Se_0.03_ screen-printed ink film, confirming the elemental composition of the fabricated sample. The EDAX analysis reveals the presence of indium (In), bismuth (Bi), tellurium (Te), and selenium (Se), validating the successful co-doping strategy. The observed atomic percentages align closely with the nominal stoichiometry, indicating effective incorporation of dopants within the InTe matrix. A minor carbon signal is also detected, likely originating from residual organic binder used in the ink formulation^36^. The inset FESEM image marks the exact region of spectrum acquisition, highlighting the analysed microstructural area. This chemical homogeneity and compositional fidelity are critical for ensuring reproducible electrical and thermoelectric behaviour in printed FTEGs.

**Fig. 5:**
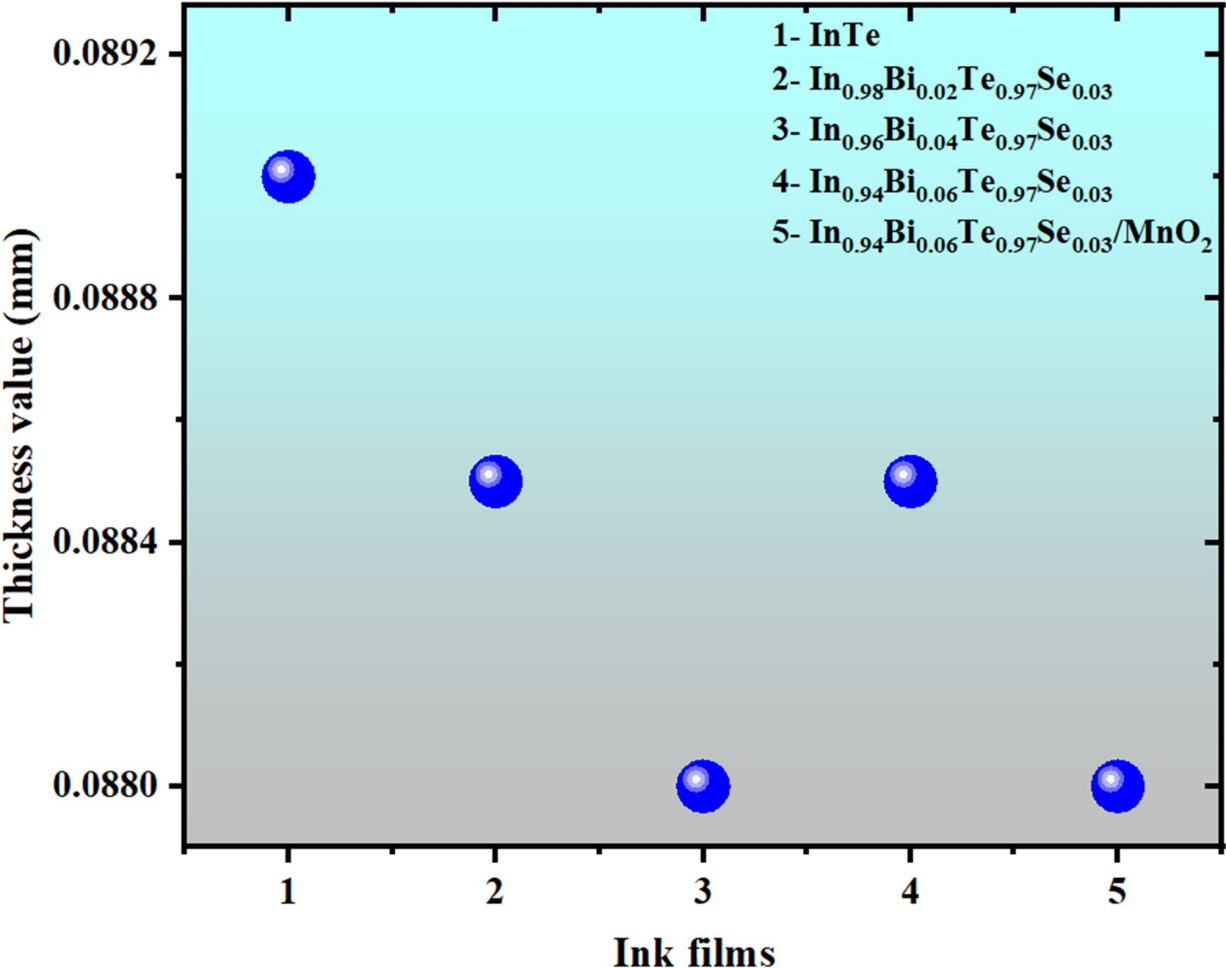
Thickness values InTe, Bi/Se co-doped InTe, and In_0.94_Bi_0.06_Te_0.97_Se_0.03_/MnO_2_ ink films.

Figure 5 displays the measured thickness values of the screen-printed InTe and Bi/Te co-doped InTe ink films. The results indicate a high degree of uniformity across multiple sample areas, with a maximum thickness variation constrained to within 2.0%. This low variability reflects the reproducibility and precision of the printing process, which is essential for ensuring consistent thermoelectric performance across devices. Thickness measurements were carried out using a high-accuracy Mitutoyo 547 − 301 digimatic thickness gauge. Based on our previous studies^26^ this contact-based measurement approach strongly agrees with cross-sectional FESEM image-based thickness evaluations, confirming its reliability.

### Hall effect and electrical conductivity analysis

**Fig. 6:**
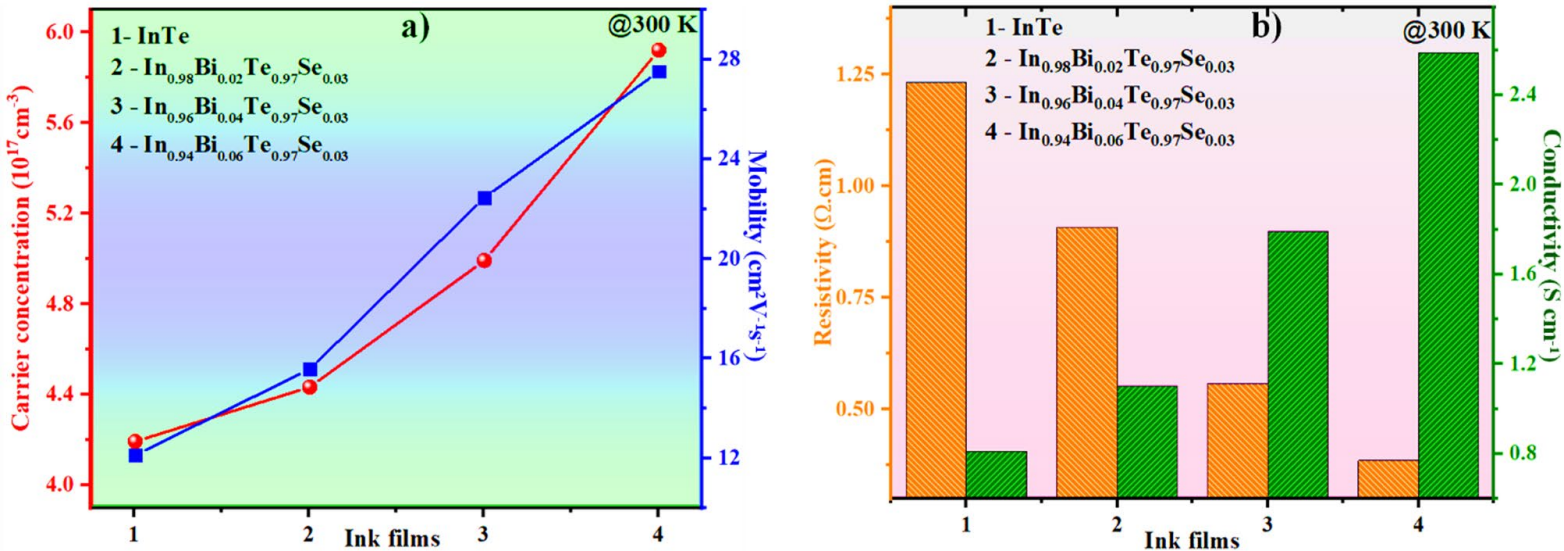
(**a**) Carrier concentration and mobility graph, (**b**) Resistivity and conductivity graph of ink samples at 300 K.

Figure 6 presents the Hall measurement results for the screen-printed pristine and co-doped InTe ink films at 300 K. Hall effect measurements performed at room temperature reveal that all ink-processed samples exhibit p-type conductivity, as indicated by the positive Hall coefficient^37^. This confirms that holes are the majority charge carriers governing electrical transport in these films^38^. Although Bi³⁺ is nominally a donor, its substitution at the In⁺ site in the p-type InTe lattice leads to an unexpected increase in hole concentration from 4.19 × 10^17^ cm^− 3^ (InTe) to 5.92 × 10^17^ cm^− 3^ (6% Bi-doped sample) (Fig. 6a). This behaviour arises from the intrinsic defect chemistry of InTe, which exhibits mixed-valence states and is prone to intrinsic indium vacancies (*V*_*In*_) that act as acceptor centres. Substitution of Bi³⁺ at the In⁺ site induces a local charge imbalance, which destabilises the lattice and promotes the formation of additional indium vacancies *(V*_*In*_*)* to restore charge neutrality, thereby increasing the hole carrier concentration^38,39^. Concurrently, Se²⁻ substitution at Te²⁻ sites perturbs the anionic sublattice, modifying bond lengths and local electronic structure, which further facilitates vacancy formation and reinforces p-type conduction^40^.

Notably, we also observed an improved crystallinity and carrier mobility enhancement in the ink-processed samples with increasing dopant concentration, an effect absent in our bulk-synthesised counterparts^22^. From an electronic structure perspective, the introduction of Bi³⁺ modifies the density of states near the valence band edge, increasing the effective mass of holes and enhancing energy-dependent carrier transport. The resulting increase in carrier concentration is accompanied by a concurrent improvement in carrier mobility (µ), from 12.542 cm^2^V^− 1^s^− 1^ (InTe) to 27.50 cm^2^V^− 1^s^− 1^ (6% Bi-doped sample) (Fig. 6a)^38^. This distinction is likely attributed to the distinct microstructural evolution induced during the ink formulation and film processing. The screen-printing process, combined with binder and controlled thermal treatment, can lead to improved grain alignment, reduced grain boundary density, and lower defect concentration. These factors collectively reduce carrier scattering and enable higher mobility^41^.

Thus, the synergistic effect of enhanced hole concentration and improved mobility in the ink-processed InTe samples can be attributed to both the dopant chemistry and the adopted processing route. The incorporation of Bi/Se into the InTe ink matrix led to a marked reduction in electrical resistivity (Fig. 6b), attributable to the enhanced carrier concentration, mobility, and improved microstructural quality of the films. As electrical conductivity (*σ = 1/ρ*) is inversely proportional to resistivity, a progressive increase in σ was observed with increasing Bi content^38^. This trend correlates well with the increase in crystallite size and the concurrent reduction in dislocation density, suggesting that the structural refinement minimises carrier scattering and facilitates charge transport^42^. These microstructural improvements collectively support enhanced electrical transport in the Bi-doped samples, which directly contribute to enhanced thermoelectric performance by boosting the power factor and, ultimately, the output power of the flexible thermoelectric generator.

### Electrical resistance, seebeck coefficient, and power output evaluations

**Fig. 7:**
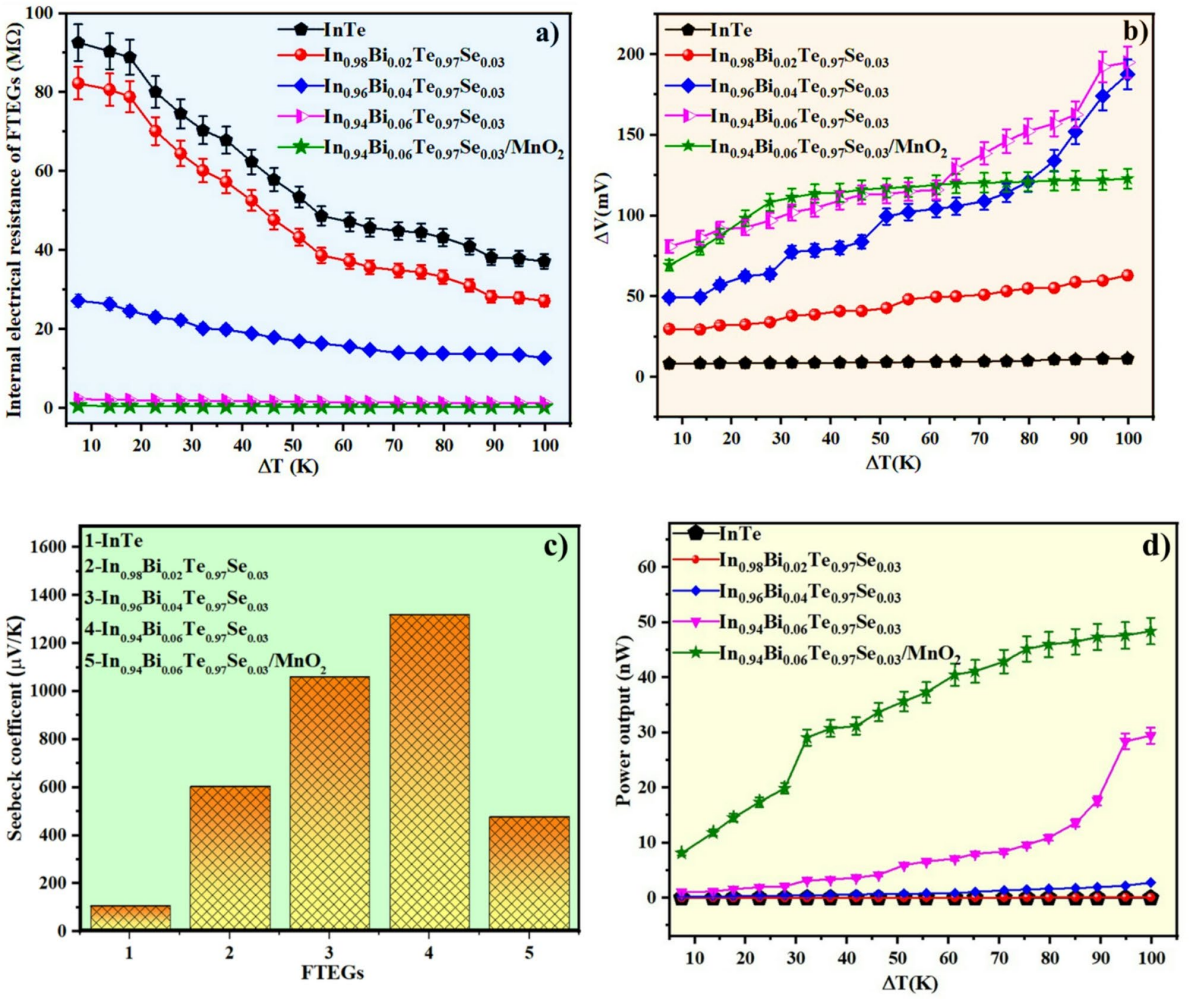
(**a**) Electrical resistance, (**b**) *∆V* Vs *∆T*, (**c**) Seebeck coefficient, (**d**) Power factor trend of prepared FTEGs.

Figure 7a displays the variation of internal electrical resistance with temperature gradient (*ΔT*) for FTEGs fabricated from pristine InTe and Bi/Se co-doped InTe inks. All devices exhibit a clear decline in resistance with increasing *ΔT*, indicating thermally activated carrier transport, a hallmark of semiconducting behaviour favourable for thermoelectric applications^43^. Among the samples, the pristine InTe shows the highest resistance across the entire *ΔT* range, owing to its relatively lower carrier concentration and limited charge transport pathways. With increasing Bi doping from 2% to 6%, the internal resistance of the FTEGs decreases significantly, from approximately 37.2 MΩ for pristine InTe to around 1.29 MΩ at *ΔT* = 100 K for the In_0.94_Bi_0.06_Te_0.97_Se_0.03_ composition, indicating a nearly 29-fold reduction in internal electrical resistance. The high resistance is mainly attributed to the insulating organic binder required for screen printing of the films. It is noted that the measured internal resistance includes a minor contribution from contact resistance (~ 2–4 Ω), which is negligible compared to the overall device resistance. This trend is attributed to improved carrier concentration, mobility, and enhanced crystallinity, as supported by increased crystallite size and reduced defect density observed in structural analyses.

Among the co-doped compositions, In_0.94_Bi_0.06_Te_0.97_Se_0.03_ exhibited the most promising electrical performance, attributed to improved mobility and microstructural refinement. Bi doping enhances crystallite growth and grain connectivity, while Se aids band tuning and indium vacancy formation, boosting hole concentration. To further reduce internal resistance, MnO₂, an n-type material previously validated in our work^26^ for its high conductivity and thermoelectric compatibility, was incorporated into the p-type matrix. Its inclusion introduces additional conduction pathways and promotes interfacial transport, yielding the lowest resistance (0.312 MΩ at *ΔT* = 100 K ) among all compositions and positioning the In_0.94_Bi_0.06_Te_0.97_Se_0.03_ /MnO₂ composite as an optimal FTEG material.

Figure 7b shows the variation of internal voltage (*ΔV*) with temperature gradient (*ΔT*) for the fabricated FTEGs. All devices demonstrate a steady increase in *ΔV* with rising *ΔT*, consistent with the thermoelectric behaviour of the samples. Among them, the In_0.94_Bi_0.06_Te_0.97_Se_0.03_ FTEG exhibits the highest *ΔV* (195 mV at *ΔT* = 100 K), followed closely by the MnO₂-composited version (123 mV), both significantly outperforming pristine InTe (12 mV). Figure 7c presents the Seebeck coefficients derived from the slopes of *ΔV* Vs *ΔT* plots. These values represent effective device-level Seebeck coefficients extracted under an applied temperature gradient, rather than intrinsic bulk room-temperature Seebeck coefficients. The pristine InTe FTEG shows the lowest Seebeck coefficient (104 µV/K), while the co-doped sample In_0.94_Bi_0.06_Te_0.97_Se_0.03_ achieves the highest value (1320 µV/K), reflecting a ~ 12.7 times enhancement. The measured *∆V* and corresponding Seebeck coefficients of the FTEGs reveal a clear improvement in thermoelectric performance with increasing Bi content and optimised co-doping. Pristine InTe shows low *∆V* and Seebeck coefficient with low carrier concentration. The potential contribution from contact thermovoltage is negligible, as the Seebeck coefficient of silver (~ 3 µV/K) is extremely low. This deviation from the typical Pisarenko trend is attributed to its poor carrier mobility, high defect density, and inefficient band alignment, which limit energy-dependent carrier transport despite the low carrier density^44^. In contrast, progressive Bi/Se co-doping enhances the electronic band structure, promotes indium-vacancy formation, and improves grain connectivity, leading to substantially higher Seebeck coefficients. This improvement originates from several intertwined mechanisms: (i) Bi-induced modification of the valence-band curvature via strong spin–orbit coupling, which increases the density-of-states effective mass and (ii) enhanced point-defect scattering from In/Bi/Se/Te mass mismatch, which introduces an energy-filtering effect that selectively transports higher-energy carriers. These combined effects significantly strengthen the Seebeck response in co-doped InTe. Moreover, the incorporation of MnO₂ into the Bi/Se co-doped InTe matrix achieved the *∆V* of 123 mV by establishing complementary conduction pathways across the p–n junction. Although the output voltage is slightly lower than that of the 6% Bi-doped sample (195 mV), the composite exhibits improved charge carrier separation due to built-in electric fields at the heterojunction interface. The slight reduction in *∆V* may be attributed to increased carrier scattering or energy filtering at the p–n interface, which can suppress low-energy carrier transport^45^. Despite this, In_0.94_Bi_0.06_Te_0.97_Se_0.03_/MnO_2_ maintains a moderate Seebeck coefficient of 476 µV/K.

The power output (*P*) of the printed flexible thermoelectric generator was derived using Eq. (4)^25^:

4 $$\:P=\frac{{V}^{2}}{R}$$

where *V* denotes voltage across the device in V, and *R* denotes its internal electrical resistance in ohms.

Figure 7d represents the power output of FTEGs as a function of *ΔT*, demonstrating a remarkable enhancement with optimised doping and composite engineering. The pristine InTe FTEG delivers a low power output of approximately 0.0034 nW at a *ΔT* of 100 K. However, with increasing Bi content, a significant improvement is observed. In particular, the In_0.94_Bi_0.06_Te_0.97_Se_0.03_ FTEG achieves a power output of around 29.45 nW. This enhancement can be attributed to synergistic effects from co-doping. Bi doping enhances carrier mobility and improves grain connectivity, while Se-mediated tuning of the band structure and vacancy formation, which together improve the Seebeck coefficient and reduce charge carrier scattering.

Further incorporation of MnO₂ into the In_0.94_Bi_0.06_Te_0.97_Se_0.03_ matrix results in a substantial power output of approximately 48.41 nW, almost 1.64 times higher than that of In_0.94_Bi_0.06_Te_0.97_Se_0.03_ FTEG. This additional boost is primarily due to the role of MnO₂ as an n-type component that forms localised p–n heterojunctions with the p-type InTe matrix, facilitating more effective electrical transport. These junctions, combined with reduced internal resistance and improved electrical pathways, contribute significantly to enhanced electron transport within the composite. Moreover, the improved interfacial contact and the formation of complementary conduction paths aid in minimising carrier recombination and maximising charge extraction^46^. Although the Seebeck coefficient of the composite is slightly lower than that of the 6% Bi-doped sample, the substantially improved electrical transport properties dominate, leading to the highest overall power output. However, the absolute power output values remain modest primarily due to the very high internal resistance of the printed films (in the MΩ range), which limits current flow and suppresses the maximum achievable output and prevents direct load-dependent power measurements under the present experimental setup. Nevertheless, the relative improvement achieved through Bi/Se co-doping and MnO₂ addition clearly demonstrates the effectiveness of our material and ink-engineering strategy.

Although the absolute power output values are modest, they fall within the expected range for printed flexible thermoelectric generators. As summarised in Table 2, previously reported printed FTEGs, fabricated using different substrates, printing conditions, and *ΔT* environments, also show power outputs in the nanowatt range. This variability reflects differences in leg dimensions, contact configuration, substrate thermal conductivity, and ink formulation. Therefore, our results are consistent with the performance trends observed in the literature for flexible printed TEGs.

**Table 2:** Power output comparison between the present work and reported thermoelectric Materials.

Compositions	Fabrication route	Substrate	Number of leg couples	Seebeck coefficient (µV/K)	Power output (nW)	∆T (K)	Ref
PEDOT: PSS/Cu_x_Se_y_ NW	vacuum-assisted filtration + cold-pressing	Nylon	9	50.8	320	30	^47^
Sb_2_Te_3_/Bi_2_Te_3_	Screen printing	Kapton	4	282	195	20	^48^
PEDOT: PSS	Solution coating	Polyester fabric	5	18.5	12.29	75	^49^
CuI/GZO	Thermal evaporation	Kaoton C*S*	17	–	10.83	293	^50^
Ag_2_Se/PVDF	physical mixing + Cold pressing	PI	5	95.9	5	30	^51^
Graphene	Printing	Paper	5	−21.5	1.7	60	^52^
AuCl_3_-doped P3HT	Spin coating	Flip film	20	163	1.9	10	^53^
Cu_2_Se	Magneton sputtering + thermal evaporation	Kapton	10	55	3.3	38	^54^
CuI	Sputtering	PET	1	–	8.2	10.8	^55^
InGaZnO	RF magnetic sputtering	PEN	2	–	0.12	53	^56^
Ni-Ag	Thermal evaporation	Silica fiber	7	–	2	6.6	^57^
Cu_2_S/PEDOT: PSS	Vacuum filtration	PI	4	38.23	23	30	^58^
SnSe/PEDOT: PSS/MWCNT/DMSO	Vacuum filtration	PI	5	22.3	14.7	39	^59^
In_0.94_Bi_0.06_Te_0.97_Se_0.03_/MnO_2_	Screen printing	PET film	8	476.0	48.41	100	*

*Present work.

### Electronic thermal conductivity studies

The electronic thermal conductivity (*K*_*e*_) of the fabricated FTEGs was calculated using the Wiedemann–Franz law, *K*_*e*_ = *LσT*^60^, where *L* is the Lorentz number (2.45 × 10^− 8^WΩK^− 2^), *σ* is the electrical conductivity, and *T* is the absolute temperature. The σ(T) values used for *K*_*e*_ calculation were obtained from direct resistivity measurements at each temperature, ensuring accurate temperature-dependent *K*_*e*_ estimation.

**Fig. 8:**
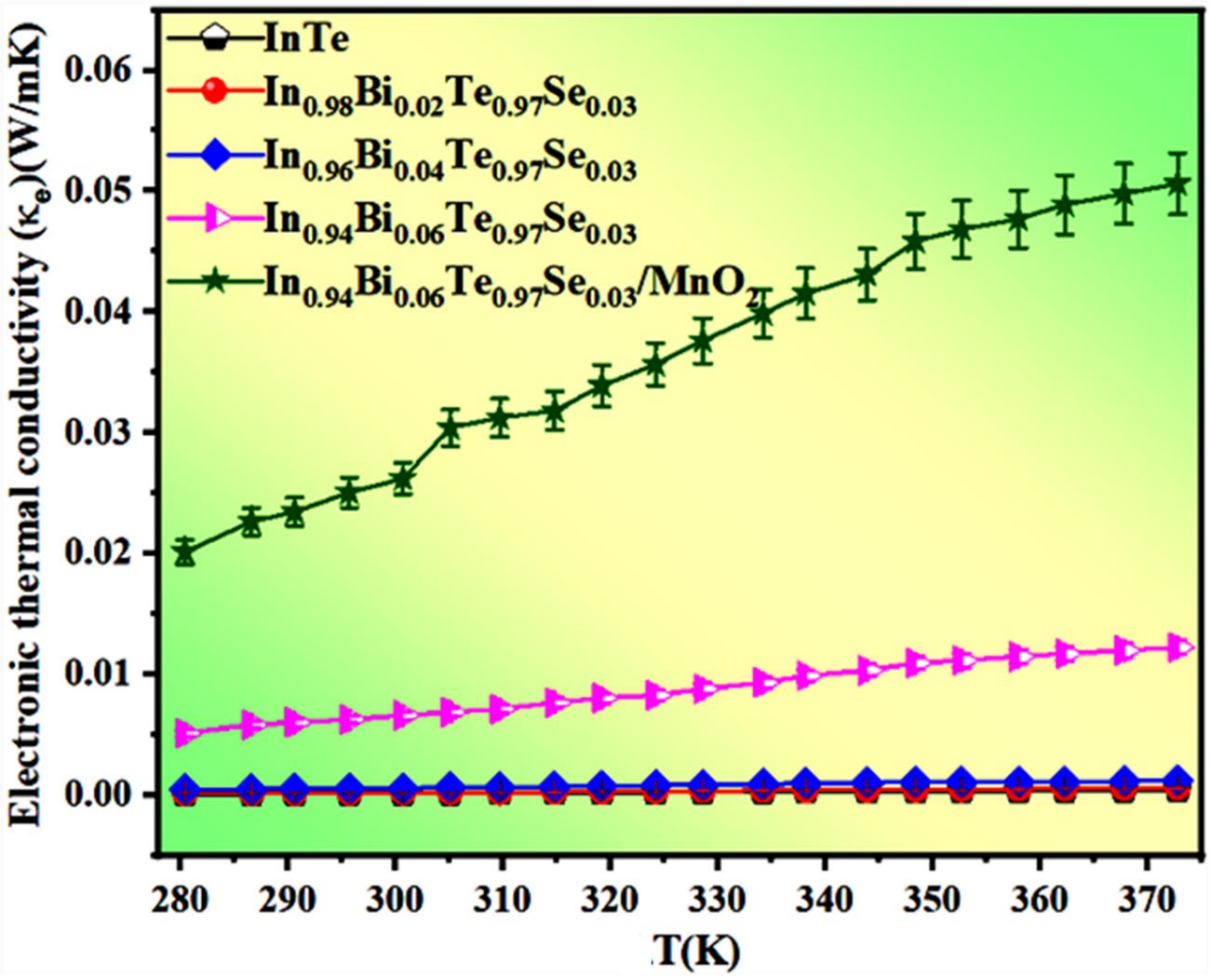
Electronic thermal conductivity (κ_e_) plot of fabricated FTEGs.

As shown in Fig. 8, *K*_*e*_ increases with temperature for all FTEGs due to thermally activated charge carriers. Among all, In_0.94_Bi_0.06_Te_0.97_Se_0.03_/MnO_2_ composite FTEG exhibits the highest *K*_*e*_, reaching ~ 0.052 W/mK at T = 373 K, reflecting its enhanced electrical conductivity and reduced internal resistance. It is noted that this analysis is limited to the electronic contribution to thermal transport. While the lattice thermal conductivity (κ_lat_) is expected to dominate in InTe, it was not measured in the present study due to experimental limitations associated with the printed flexible films. Therefore, no conclusions regarding total thermal conductivity or ZT are drawn here.

### Flexibility assessment of FTEGs

**Fig. 9:**
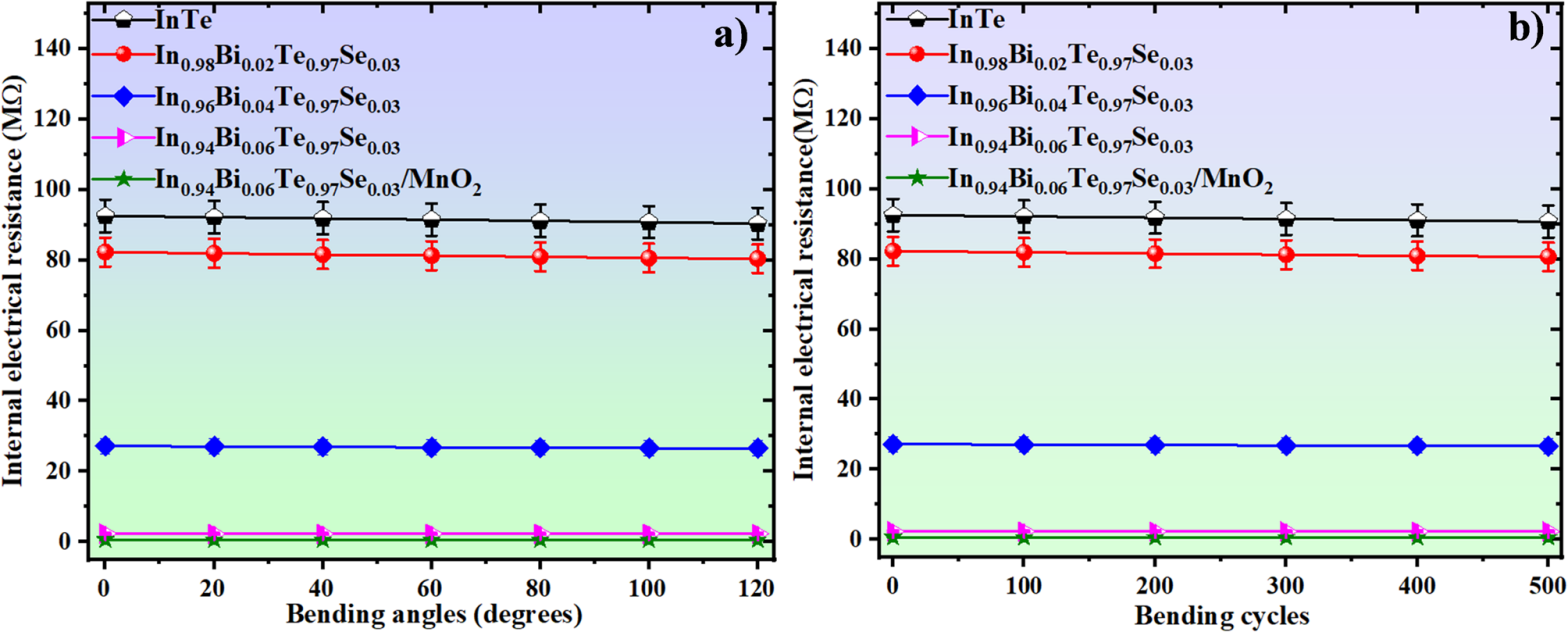
(**a**) Internal resistance Vs bending angle, (**b**) Internal resistance Vs bending cycles of fabricated FTEGs.

The mechanical flexibility and operational stability of the screen-printed FTEGs were thoroughly assessed through bending angle and cyclic bending tests, which are critical for evaluating their applicability in real-world flexible electronics^61^. Electrical resistance measurements under static bending angles of 0°, 30°, 60°, 90°, and 120° revealed a modest decrease of approximately 2% (Fig. 9a). This slight reduction in electrical resistance suggests that the device structure accommodates mechanical strain without degrading electrical performance, indicating robust mechanical-electrical coupling in the printed films.

Furthermore, durability under repeated mechanical stress was examined by subjecting the devices to 500 bending cycles (0, 100, 200, 300, 400, 500 cycles). The resistance again showed a small yet consistent decrease of about 2% over the entire cycling process (Fig. 9b), which is less compared to other published works^50^. From a physical standpoint, this mechanical endurance reflects strong interfacial adhesion between the active material and the flexible substrate, facilitated by the screen-printing process and the polymer binder system. The minimal change in resistance over many cycles indicates that the film microstructure remains intact and free from significant crack propagation or delamination, common failure mechanisms in brittle thermoelectric films.

These results emphasise the mechanical robustness and structural resilience of the FTEGs, which retain their thermoelectric functionality even under continuous flexural stress. This is especially critical in wearable and portable applications where devices experience dynamic movements. The minor resistance variation suggests stable electrical connectivity and confirms the effective integration of mechanical flexibility with efficient charge transport. Thus, these FTEGs offer a promising platform for next-generation energy harvesting systems in soft and deformable electronics.

## Conclusion

This work presents a pioneering demonstration of flexible thermoelectric generators (FTEGs) based on InTe, synthesised via a conventional solid-state reaction route and fabricated through a scalable screen-printing process. Structural analysis confirmed a phase-pure tetragonal InTe lattice, with Bi/Se co-doping producing a systematic increase in crystallite size and a reduction in dislocation density and microstrain. These improvements were supported by FESEM observations, which showed enhanced film compactness and grain connectivity in highly doped samples. Thermoelectric measurements revealed that Bi/Se co-doping substantially boosts device performance. The In_0.94_Bi_0.06_Te_0.97_Se_0.03_ sample exhibited the highest Seebeck coefficient (~ 1320 µV/K), *ΔV* (~ 195 mV at *ΔT* = 100 K), and a significantly reduced internal resistance (~ 1.29 MΩ), resulting in a power output of ~ 29.45 nW, nearly 29 times higher than that of pristine InTe. These enhancements stem from synergistic effects such as increased carrier concentration from Bi-induced indium vacancies, improved grain connectivity by Bi/Se.

Further enhancement was achieved by introducing MnO₂ as a complementary conductive additive, forming local p–n-type heterojunctions that facilitated efficient electrical transport. This composite achieved the highest power output (~ 48.41 nW) with a significantly reduced internal resistance (~ 0.312 MΩ), despite a lower Seebeck coefficient. The printed FTEGs also exhibited excellent flexibility, with only ~ 2% resistance variation after 500 bending cycles and bending angles up to 120°, confirming their mechanical durability. This combination of flexibility, power output, and low fabrication cost makes InTe-based printed FTEGs promising candidates for future wearable and on-body energy-harvesting systems. Overall, this study establishes InTe as a promising platform for printable thermoelectrics. The combined effects of Bi/Se co-doping and n-type MnO₂ incorporation effectively modulate structural characteristics, improve carrier transport, and enhance power generation capability. Future work will focus on optimising ink formulations, analysing lattice thermal conductivity, and developing multilayer or patterned device architectures to further advance the performance of flexible thermoelectric systems.

## Data Availability

The datasets used and analysed during the current study are available from the corresponding author on reasonable request.

## Declarations

### Statement on the use of generative AI

Grammarly and ChatGPT were employed exclusively as language assistance tools to paraphrase and improve the clarity of the manuscript, without affecting the originality, scientific content, or data interpretation.
